# Translating a gene expression signature for multiple myeloma prognosis into a robust high-throughput assay for clinical use

**DOI:** 10.1186/1755-8794-7-25

**Published:** 2014-05-17

**Authors:** Ryan van Laar, Rachel Flinchum, Nathan Brown, Joseph Ramsey, Sam Riccitelli, Christoph Heuck, Bart Barlogie, John D Shaughnessy Jr

**Affiliations:** 1 Signal Genetics, 667 Madison Avenue, 14th Floor, New York, NY 10065, USA; 2 University of Arkansas for Medical Sciences, Myeloma Institute for Research and Treatment, 4301 West Markham, Slot 816, Little Rock, AR 72205, USA

## Abstract

**Background:**

Widespread adoption of genomic technologies in the management of heterogeneous indications, including Multiple Myeloma, has been hindered by concern over variation between published gene expression signatures, difficulty in physician interpretation and the challenge of obtaining sufficient genetic material from limited patient specimens.

**Methods:**

Since 2006, the 70-gene prognostic signature, developed by the University of Arkansas for Medical Sciences (UAMS) has been applied to over 4,700 patients in studies performed in 4 countries and described in 17 peer-reviewed publications. Analysis of control sample and quality control data compiled over a 12-month period was performed.

**Results:**

Over a 12 month period, the 70-gene prognosis score (range 0–100) of our multiple myeloma cell-line control sample had a standard deviation of 2.72 and a coefficient of variance of 0.03. The whole-genome microarray profile used to calculate a patient’s GEP70 score can be generated with as little as 15 ng of total RNA; approximately 30,000 CD-138+ plasma cells. Results from each GEP70 analysis are presented as either low (70-gene score <45.2) or high (≥45.2) risk for relapse (newly diagnosed setting) or shorter overall survival (relapse setting). A personalized and outcome-annotated gene expression heat map is provided to assist in the clinical interpretation of the result.

**Conclusions:**

The 70-gene assay, commercialized under the name ‘MyPRS®’ (Myeloma Prognostic Risk Score) and performed in Signal Genetics’ CLIA-certified high throughput flow-cytometry and molecular profiling laboratory is a reproducible and standardized method of multiple myeloma prognostication.

## Background

By coupling immunomagnetic and fluorescence-based cell separation with microarray gene expression profiling, researchers have dramatically improved the understanding of how hematological malignancies, including Multiple Myeloma (MM), develop, progress, and respond to therapy. Multiple Myeloma accounts for 1% of all cancers, affecting an estimated 22,350 people in the US in 2013 and resulting in 10,710 deaths (cancer.gov). Gene expression signatures, generated using tissue obtained at the time of diagnosis, have been demonstrated to accurately predict patient outcome and stratify patients into clinically relevant molecular subgroups in many types of cancers [1-5].

By performing large multidisciplinary studies of multiple myeloma, researchers at University of Arkansas for Medical Sciences (UAMS) developed a 70-gene signature of aggressive disease (GEP70), corresponding to increased risk of relapse and poorer overall survival probability [6]. This signature was independently validated in separate patient populations for its ability to predict risk of relapse and shorter overall survival in newly diagnosed multiple myeloma and proved superior to other prognostic risk scores in multivariate analyses. In the post-relapse setting, GEP70 is able to stratify patients into groups with highly significant differences in overall survival [7]. Since 2006, the UAMS GEP70 assay has been validated on patient cohorts totaling over 4,700 patients, described in the 17 publications listed in Table 1. These validation studies, performed independently by German, French, Italian, British, Dutch, and US-based clinical research groups, have repeatedly shown that the prognostic significance the 70-gene algorithm is superior to both conventional risk stratification methods and other gene expression signatures in multivariate analyses. Patients identified as high risk by GEP70 (ranging from 15-30% of all patients, depending on the characteristics of the patient population profiled) may benefit from alternative treatment regimens and/or referral to an appropriate clinical trial. Importantly, the vast majority of cases, defined as low risk, might benefit from reduced intensity treatments.

**Table 1:** Peer-reviewed publications describing the use of GEP70/MyPRS® gene expression profiling on patients with multiple myeloma

**Date**	**No. patients**	**Patient series**	**Publication**
1-Jan-2006	351	Newly diagnosed patients with MM treated with 2 cycles of high-dose melphalan and autologous stem cell transplantation [8].	Shaughnessy JD Jr, Barlogie B. “Using genomics to identify high-risk Myeloma after autologous stem cell transplantation”. Biol Blood Marrow Transplant 2006; 12 (1 Suppl 1):77–80.
25-May-2006	414	Newly diagnosed patients treated with high-dose melphalan-based tandem transplants [9].	Zhan et al. “The molecular classification of multiple myeloma”. Blood 2006; 108(6):2020–2028.
14-Nov-2006	532	Newly diagnosed patients with multiple myeloma (MM) treated on 2 separate protocols [6].	Shaughnessy et al. “A validated gene expression model of high-risk multiple myeloma is defined by deregulated expression of genes mapping to chromosome 1”. Blood 2007; 109:2276–84.
9-May-2007	220	Newly diagnosed patients treated with TT2 [10].	Shaughnessy et al. “Testing standard and genetic parameters in 220 patients with multiple Myeloma with complete data sets: superiority of molecular genetics”. Br J Haematol 2007; 137:530–536.
22-Jun-2007	303	Newly diagnosed patients with myeloma treated with Total therapy 3 (incorporating bortezomib into a melphalan-based tandem transplant regimen) [11].	Barlogie et al. “Incorporating bortezomib into upfront treatment for multiple myeloma: early results of total therapy 3”. Br J Haemotol 2007; 138:176-185
7-Sep-2007	71	Newly diagnosed multiple myeloma patients treated with high-dose melphalan and stem cell transplant [12].	Chng et al. “Translocation t(4;14) retains prognostic significance even in the setting of high-risk molecular signature”. Leukemia 2008; 22:459–61.
1-Dec-2007	326	Newly diagnosed patients with myeloma received a tandem autotransplant regimen [13].	Haessler et al. “Benefit of complete response in multiple myeloma limited to high-risk subgroup identified by gene expression profiling”. Clin Cancer Res. 2007; 13(23):7073-7079
15-Jan-2008	156	Relapsed myeloma patients enrolled in the APEX phase 3 clinical trial that compared single-agent bortezomib (B) to high-dose dexamethasone (HD) [14].	Zhan et al. “High-risk myeloma: a gene expression based risk-stratification model for newly diagnosed multiple myeloma treated with high-dose therapy is predictive of outcome in relapsed disease treated with single-agent bortezomib or high-dose dexamethasone.” Blood 2008; 111(2):968–969.
30-Jun-2008	250	Two hundred fifty patients with myeloma at diagnosis with at least 500,000 available bone marrow CD138+ plasma cells [15].	Decaux etl al. Prediction of Survival in Multiple Myeloma Based on Gene Expression Profiles Reveals Cell Cycle and Chromosomal Instability Signatures in High-Risk Patients and Hyperdiploid Signatures in Low-Risk Patients: A Study of the Intergroupe Francophone du Myélome JCO October 10, 2008:4798–4805;
29-Mar-2009	290	Untreated myeloma patients with cytogenetic abnormalities (CA) present in randomly sampled (RS) or focal lesion (FL) bone marrow sites [16].	Zhou et al. “Cytogenetic abnormalities in multiple myeloma: poor prognosis is linked to concomitant detection in random and focal lesion bone marrow samples and associated with high-risk gene expression profile”. Br J Haematol 2009; 145(5):637-641
25-Jun-2009	120	Myeloma patients previously enrolled in tandem transplantation trial Total Therapy 2 [7].	Nair et al. “Gene expression profiling of plasma cells at myeloma relapse from tandem transplantation trial Total Therapy 2 predicts subsequent survival”. Blood 2009; 113:6572–5.
14-Mar-2010	258	Newly diagnosed patients with multiple myeloma entered into the MRC Myeloma IX study [17].	Dickens et al. Homozygous Deletion Mapping in Myeloma Samples Identifies Genes and an Expression Signature Relevant to Pathogenesis and Outcome. Clin Cancer Res March 15, 2010 16:1856–1864;
12-Apr-2010	52	Patients newly diagnosed with MM [18].	Zhou et al. “High-risk myeloma is associated with global elevation of MiRNAs and overexpression of EIF2C2/AGO2”. Proc Natl Acad Sci USA 2010; 107(17): 7904-790
30-Sep-2010	757	Previously untreated patients undergoing high-dose chemotherapy [19].	Hose et al. “Proliferation is a central independent prognostic factor and target for personalized and risk adapted treatment in multiple myeloma”. Haematologica 2011; 96(1):87–95.
20-Aug-2010	275	Newly diagnosed patients with symptomatic or progressive myeloma [20].	van Rhee et al. Total Therapy 3 for multiple myeloma: prognostic implications of cumulative dosing and premature discontinuation of VTD maintenance components, bortezomib, thalidomide, and dexamethasone, relevant to all phases of therapy. Blood 2010 116:1220–1227;
7-Oct-2010	320	Newly diagnosed patients with MM (Dutch-Belgian Cooperative Trial Group for Hemato-Oncology [21].	Broyl et al. Gene expression profiling for molecular classification of multiple myeloma in newly diagnosed patients. Blood 2010 116:2543–2553;
22-Aug-2011	45	Patients with myeloma receiving initial therapy with lenalidomide and dexamethasone [22].	Kumar et al. “Impact of gene expression profiling-based risk stratification in patients with myeloma receiving initial therapy with lenalidomide and dexamethasone”. Blood 2011; 118(16): 4359–4362.

Publications listed are the first use of GEP70 to stratify patients in the relevant cohort as high or low risk for. Additional publications reanalyzing the same (or subsets of a) patient series are not shown.

In order to translate any gene expression signature from the research setting to routine use in a clinical laboratory, a number of logistical and technical challenges must be overcome. These include defining the minimum amount of patient specimen (e.g. bone marrow aspirate) required to isolate sufficient plasma cell RNA for expression profiling and establishing a comprehensive quality control framework in order to monitor laboratory performance over time and ensure reliability of results. Yet another challenge is how best to present the gene expression algorithm results in order to enable straightforward interpretation by treating physicians and incorporation into patient management regimens.

In this paper we describe the use of a high-throughput process, combining cell isolation, flow cytometry and gene expression profiling to provide physicians with personalized prognostic assessments of multiple myeloma, using bone marrow aspirate, based on the comprehensively validated GEP70 signature. Data are presented to describe the stability of the assay over time as performed in a CLIA-certified clinical laboratory diagnostic setting.

## Methods

### Plasma cell quantification and separation

Processing of bone marrow aspirate specimens submitted for MyPRS® analysis occurs largely as previously described [23]. CD138+ plasma cell isolation from red blood cell lysed bone marrow aspirates is performed by immunomagnetic bead selection with monoclonal mouse antihuman CD138 antibodies using the AutoMACS Pro automated separation system (Miltenyi-Biotec, Auburn, CA). Minimum PC purity of ≥80% homogeneity is confirmed by 2-color flow cytometry using CD38+/CD45− post-sort (after immunomagnetic bead selection) criteria (Becton Dickinson, San Jose, CA).

Determination of CD138+ cell presence is performed on the initial whole bone marrow aspirate by removing an aliquot from the gently homogenized bone marrow aspirate that was mixed with EDTA at the time of collection. This aliquot is incubated with CD138 PE and CD45 FITC antibodies (Miltenyi, CA), and then the red blood cells are lysed. The remaining cells are washed with phosphate buffered saline (PBS) and flow cytometry is performed. The pre-sort cell percentage (prior to immunomagnetic bead selection) is determined by identifying the CD138+/CD45- cells from the total population after red blood cell (RBC) lysis. This determination is performed on either the FACS Calibur system or the FACS Aria III system (Becton Dickinson, NJ). Once the presence of CD138+ cells has been confirmed, the bone marrow aspirate undergoes RBC-lysis and is washed with autoMACS Running Buffer (Miltenyi, CA).

Cell count is determined using the Nucleocounter NC-100 (Chemometec, Denmark) according to manufacturer recommendations. Immunomagnetic beads are then bound to the cells and the remaining unbound beads are removed through a second Running Buffer wash. The CD138+ cells are isolated from the remaining cells using the AutoMACS Pro Separator (Miltenyi, CA) according to manufacturer recommendations. If 80% cell homogeneity is not obtained, the specimen either undergoes a second immunomagnetic isolation and/or enriched using CD38 PE and CD45 FITC antibodies on the FACS Aria III (Becton Dickinson, NJ).

In keeping with institutional, federal, and Helsinki Declaration guidelines, all identifiable patients gave written informed consent for undergoing bone marrow sampling for gene expression profiling and the institutional review board of the University of Arkansas for Medical Sciences approved the research studies. Consent was not obtained from patients where data were analyzed anonymously and not associated with any identifiable or longitudinal information.

### RNA isolation and microarray analysis

Cell lysis and total-RNA isolation from purified CD138+ plasma cells is performed using the RNeasy Micro Kit (Qiagen, Germany). RNA concentration and purity is determined using a Nanodrop Spectrophotometer (Thermo Scientific, Wilmington) and the integrity is assessed using the Agilent Bioanalyzer 2100 system (CA). Double-stranded complementary DNA (cDNA) and amplified biotinylated antisense RNA (aRNA or cRNA) are synthesized from total RNA using the Affymetrix 3′ IVT Express Kit. The aRNA is fragmented and hybridized to whole-genome U133 Plus 2.0 GeneChip microarrays (Affymetrix, Santa Clara, CA), according to manufacturer recommendations. Hybridized GeneChips are scanned with the Affymetrix GeneChip Scanner 3000DX V2, an FDA-cleared, CE-IVD marked system. Scanned GeneChip files (CEL files) are normalized and assessed for hybridization success and sample quality by a proprietary gene expression data quality control system (ResultsPX™), previously described [2].

GEP70 risk scores are calculated using the method originally described by Shaughnessy et al. [6], with the additional modification of scaling the score to a range of 0 to 100 to assist in interpretation. This scaling is done using the equation [original GEP70 + 1.6] * 20 = scaled GEP70.

### Control sample analysis

Positive and negative control specimens are analyzed alongside all clinical MyPRS® samples. Positive control-sample analysis is performed using the multiple myeloma cell line H929 which is grown as recommended (American Type Culture Collection, Chantilly, VA). To prepare the cell line for repeated control sample use, cells are consolidated and analyzed for homogeneity and for CD138+ presence. Once +80% CD138+ homogeneity is confirmed, the cells are pelleted, placed on RLT (plus 2-mercaptoethanol) buffer and frozen at −80°C. Each aliquot of H929 cells is tested over several months to generate sufficient data in order to calculate the standard deviation of its GEP70 risk score. A Levy Jennings plot is used to analyze the positive control specimen processed in parallel to each batch of clinical specimens, with results outside of the median +/−3SD range being rejected.

Negative control analysis is performed using aliquots of RLT (+2-mercaptoethanol) buffer and frozen. The negative control is inserted into a batch of samples at RNA isolation and is carried all the way through aRNA amplification. Detection of aRNA in the negative control specimen prior to microarray hybridization results is a sign of contamination and cause for rejection.

### Replication of GEP70 signature between UAMS and Signal Genetics laboratories

To evaluate the difference between GEP70 risk scores generated in the original research laboratory (UAMS) and the clinical laboratory (Signal Genetics LLC, AR) a series of 99 bone marrow aspirates were analyzed. Specimen preparation, microarray hybridization and data analysis methods were performed as described above. GEP70 scores were compared by performing intra-class correlation, Passing and Bablok regression and chi-square analysis of high/low risk group classification in MedCalc 12.7.8 (MedCalc Software bvba, Belgium) [24].

### Interpretation of GEP70 score using personalized gene expression heat maps

The GEP70 risk score for each MyPRS analysis performed was visualized by creation of a personalized two-dimensional heat map generated by the ResultsPX™ genomic data management platform developed by Signal Genetics. This system uses Microsoft SQL Server (Redmond, WA) databases, R [25] and Bioconductor [26] and custom scripts to display the expression profile of the prognostic GEP70 gene score within the context of the 559 multiple myeloma patients from two previously published datasets, including data from patients used to develop the algorithm [6]. The 5-year relapse status of each patient is shown at the top of each gene profile (red: relapse, blue: no relapse) along with the corresponding risk score.

To generate a personalized gene expression data heat map for each patient analyzed, the following steps are performed by Signal Genetics ResultsPX™ analysis software:

i. Load the matrix of the MAS5 normalized gene expression data for the 70 genes by 559 patients.

ii. Attach the 70 gene data from the patient being analyzed to the matrix, making it 70 genes by 560 patients.

iii. Order 560 columns of the matrix (1 column = 1 patient) left-to-right by increasing GEP70 score.

iv. Order the 70 rows of the matrix (1 row = 1 gene) using hierarchical clustering, as implemented by ‘hclust’ R library (http://stat.ethz.ch/R-manual/R-patched/library/stats/html/hclust.html).

No ‘batch effect’ modification is performed during this procedure as the 70 gene x 559 patient dataset was that used to originally develop the GEP70 algorithm. These data were produced by the UAMS MIRT laboratory and were used in the validation studies performed herein to ensure the 70-gene assay produces statistically equivalent results when performed in the Signal Genetics clinical laboratory.

## Results

### Genomic profiling of paired aliquots of patient CD138+ cells in research and clinical laboratories shows high correlation of GEP70 scores

**Figure 1:**
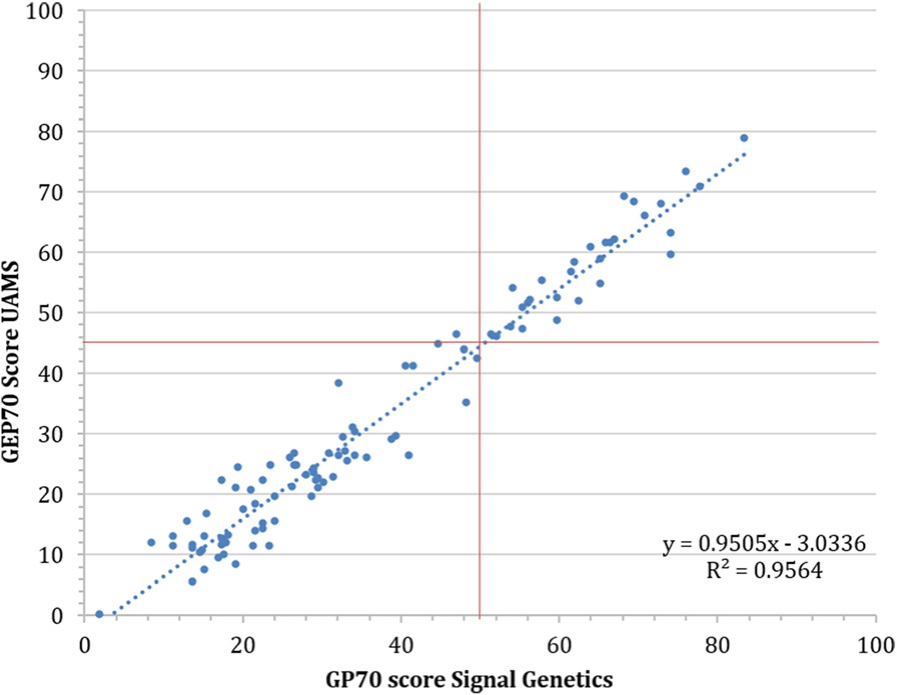
**Inter-laboratory reproduciblity; Analysis of GEP70 scores calculated on 99 clinical bone marrow aspirate specimens analyzed in parallel by UAMS Myeloma Instiute for Research and Treatment (MIRT) (1a. y-axis) and Signal Genetics CLIA laboratory (1b x-axis).** Lines at 45.2 correspond to the low/high risk threshold.

Ninety-nine patient bone marrow aspirate specimens were split into two aliquots and processed as described in both the UAMS research laboratory and at Signal Genetics’ CLIA-certified laboratory (Figure 1). The intraclass correlation coefficient for the set of 99 GEP70 scores generated by UAMS and Signal Genetics is 0.98 (95% CI: 0.97 to 0.99), indicating a high level of consistency. The Cusum test for linearity revealed no significant deviation from linearity (P = 0.17) [24].

In order to assess the clinical implications of the small difference in risk scores observed between the two laboratories, ROC analysis was performed using 5-year relapse-free survival as the binary outcome metric. The AUC of the UAMS risk score was 0.67 (95% CI 0.57 to 0.76) compared to the Signal Genetics AUC of 0.66 (95% CI: 0.56 to 0.75), a statistically insignificant difference (P = 0.402). This indicates that no significant difference exists in the association of the GEP70 score with multiple myeloma relapse risk based on the processing of a specimen in the research or clinical laboratory setting.

### Analysis of control specimen GEP70 risk score shows high level of consistency in MyPRS® analyses over time

**Figure 2:**
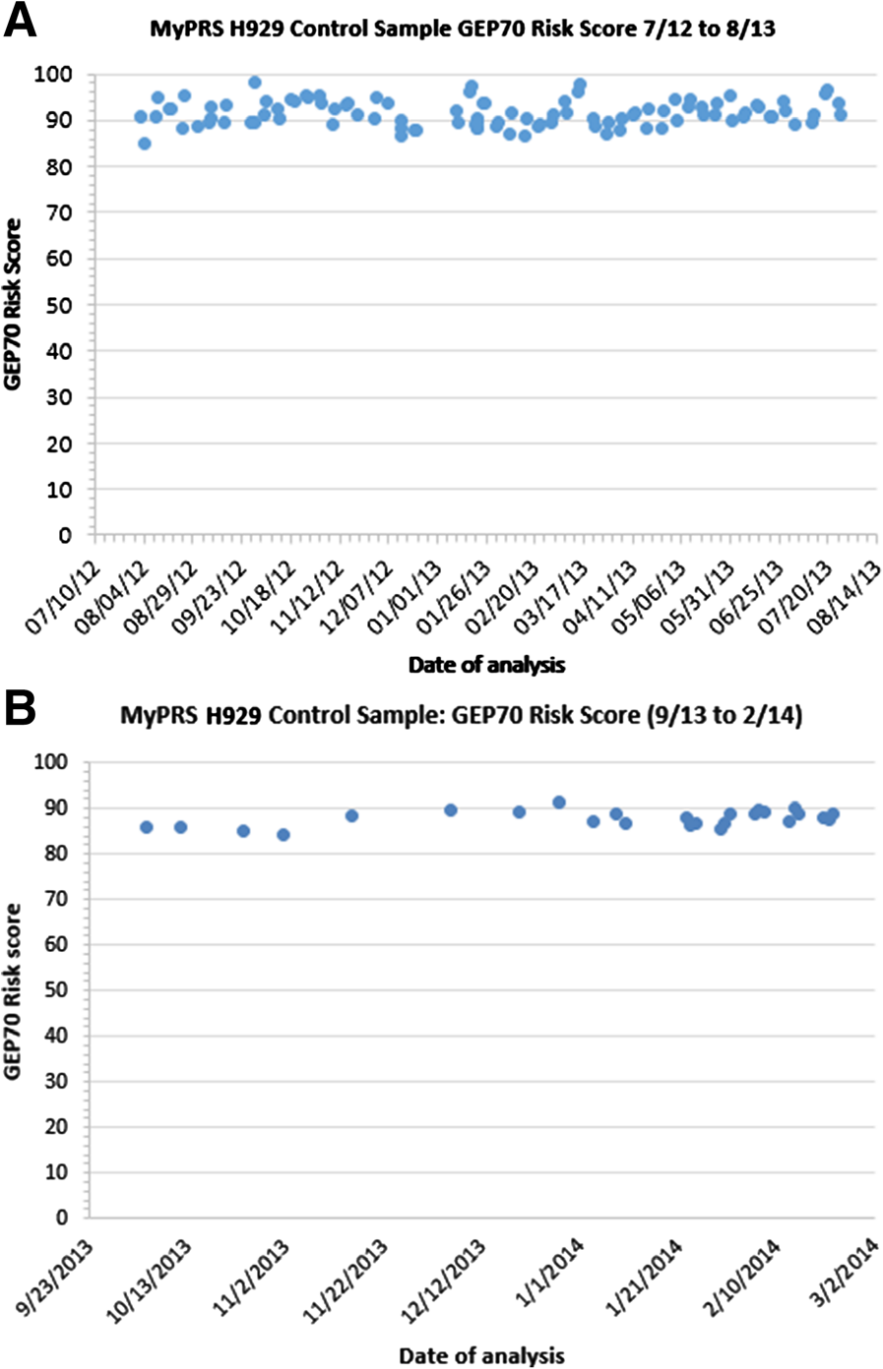
**Analysis of MyPRS Control Sample stability over time.** MyPRS Control Sample stability over time; **(A)** H929 Control sample GEP70 scores generated bewteen August 2012 and August 2013 exhitit high stability over time. No gradual shift up or down in risk score is observed. Standard deviation of risk scores in this analysies was 2.72 and a CV of 0.03. **(B)** Control sample data from September 2013 to February 2014 (new aliquot of H929) shows further improvements in assay stability. Standard deviation 1.70, CV 0.019.

Along with each batch of clinical bone marrow aspirate samples analyzed, an aliquot of RNA from a multiple myeloma cell-line (H929) is analyzed and its GEP70 score is assessed for stability. Over a twelve-month period from August 2012 to August 2013, 102 control sample analyses were performed. As shown in Figure 2, the median value of the GEP70 score over time was 91.2, with a standard deviation of 2.7 and 3.0% coefficient of variance.

**Figure 3:**
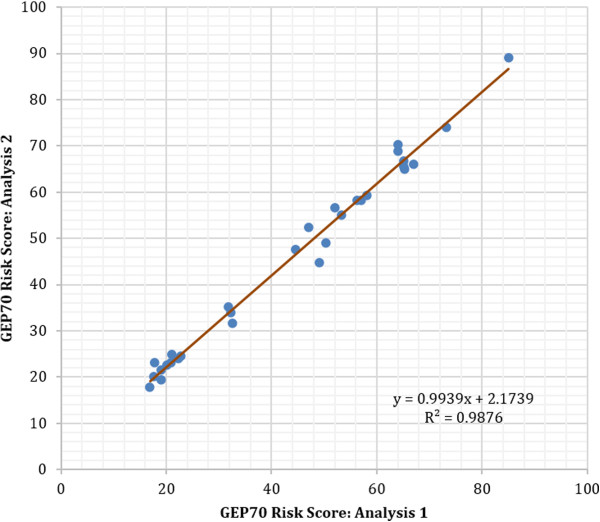
**Intra-laboratory reproducibility; Comparison of GEP70 scores from 30 specimens analyzed in duplicate.** Correlation coefficient of 0.98 shows an extremely high degree of reproducibility between experiments.

Next we sought to evaluate the reproducibility of the GEP70 scores across the dynamic range of the assay (i.e. low risk 0 to 45.2, high risk 45.2 to 100). Thirty specimens of MM RNA were analyzed in duplicate, approximately one month apart (Figure 3). A high degree of correlation was observed between the repeated measurements (r^2^ = 0.99) with no statistically significant deviation from linearity detected by the Cusum test (P = 0.98).

### Variation in clinical bone marrow aspirate specimen plasma cell content has negligible impact on RNA isolation and gene expression profile quality

Bone marrow aspirate specimens of varying absolute and relative CD138+ plasma cell content are submitted for GEP70 analysis from treatment centers throughout the United States. Immunomagnetic and fluorescence based isolation of CD138+ plasma cells is routinely performed using methods described on every specimen to isolate, and if necessary enrich, the target plasma cells in the specimen.

We investigated the association between the relative malignant cell content, RNA integrity and the resulting GEP70 risk scores by performing a retrospective analysis of data generated from routine bone marrow aspirate specimens submitted to Signal Genetics over a period of twelve months. Agilent Bioanalzyer RNA integrity number (RIN; range 0–10) and the GEP70 risk score (range 0–100) data were compiled from 1000 randomly selected specimens submitted to Signal Genetics for routine GEP70 analysis between August 2012 and July 2013.

**Figure 4:**
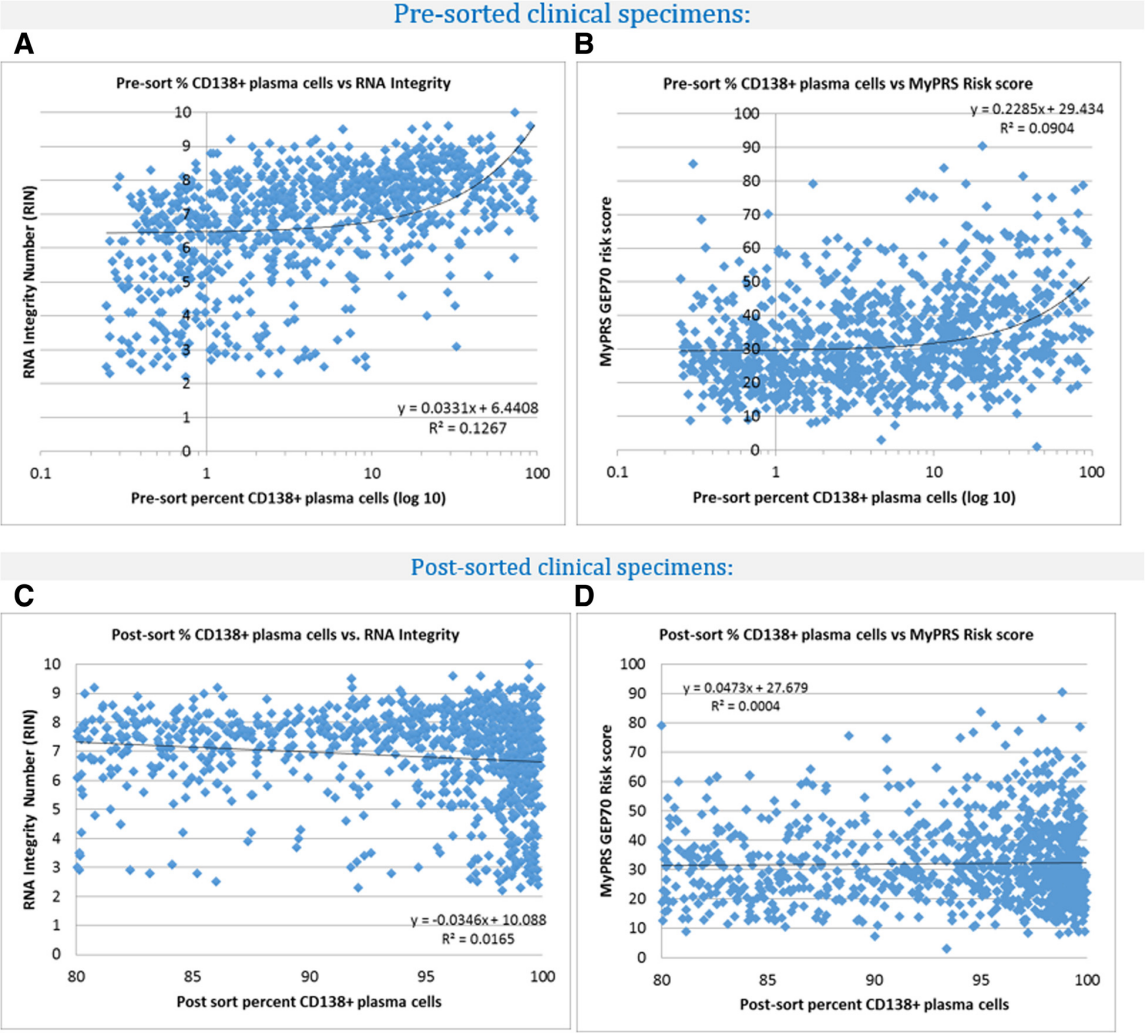
**A-D: Analysis of bone marrow aspirate specimen variability vs. RNA quality and GEP70 risk score; The relative CD138+ cell content (pre- and post- sorting) vs RNA integrity and GEP70 risk score of 1000 randomly selected clinical specimens submitted for MyPRS analysis is shown above.** The wide range in cellularity of specimens submitted for MyPRS analysis (0.25 - 96.2%) does not impact on the quality of the RNA isolated for gene expression profiling, nor the final GEP70 risk score.

Within this series of 1000 specimens, the CD138+ cell content ranged from the lower acceptance threshold of 0.25% to 96.2% (mean: 12.80%, median: 4.63%). Despite this wide range of cellularity, skewed toward the lower end of the spectrum, Figure 4a and b show that only a weak correlation exists between the RNA integrity number, GEP70 score and pre-sorted specimen percentage of CD138+ cells (r^2^ = 0.13 and 0.010, respectively). After cell sorting, specimens with less than 80% purity are excluded from further analysis in order to ensure the genomic profile represents the cells of interest rather than other potentially contaminating material. Inspection of specimens with cell content between 80 and 100% revealed there is no significant association between the relative CD138+ plasma cell content and RNA integrity, or the GEP70 risk score, r^2^ = 0.017 and r^2^ = <0.001, respectively (Figure 4c-d).

These data show that the specimen preparation methods used to isolate the malignant cells from a patient’s bone marrow aspirate are robust and not impacted by natural variations in specimen quality and relative quantity of malignant plasma cells. This ensures the GEP70 prognostic risk score is an accurate and reproducible prediction of patient prognosis, with negligible impact from biological or other sources of specimen variation.

### Determining the minimum amount of CD138+ plasma cell RNA required for reliable gene expression profiling

As stated, patient bone marrow aspirate specimens vary in terms of plasma cell number, viability and purity. To determine minimum number of viable CD138+ plasma cells necessary to generate a high quality GEP and reproducible GEP70 score, we performed two titration studies in which varying amounts of pooled MM aRNA were hybridized to Affymetrix microarrays in triplicate.

By hybridizing varying amounts of pooled aRNA (range: 10 μg to 2 μg) to multiple GeneChip we were able to extrapolate to a minimum number of CD138+ plasma cells required to accept a specimen for routine GEP70 analysis. GeneChip quality control metrics and GEP70 risk scores were used to assess the impact of the using lower amounts of aRNA compared to the amounts protocols used in previous studies [6,7,9]. After repeating the experiment twice, it was apparent that there was negligible variation in the GEP70 score, even using as little as 2ug of pooled aRNA, as shown in Table 2. The variance of the GEP70 score in titration experiment 1 was 1.5% and in experiment 2 was 2.8%.

**Table 2:** GeneChip QC metric summary and GEP70 scores for pooled aRNA titration experiments

**Titration study Experiment 1 Chip ID**	**μg aRNA hybridized to chip**	**GEP70 risk group**	**GEP70 risk score**	**Mean GEP70**	**Standard deviation**	**Chip QC Metrics Fail/Warning/Pass**
RE13-000031-850291.CEL	10	Low	41.11	41.73	0.56	0/0/7
RE13-000032-850251.CEL	10	Low	42.19			0/0/7
RE13-000033-850371.CEL	10	Low	41.88			0/0/7
RE13-000034-850240.CEL	8	Low	41.20	41.25	0.29	0/0/7
RE13-000035-850377.CEL	8	Low	40.98			0/0/7
RE13-000036-850294.CEL	8	Low	41.55			0/0/7
RE13-000037-850370.CEL	6	Low	40.67	41.31	1.01	0/0/7
RE13-000038-850379.CEL	6	Low	42.47			0/0/7
RE13-000039-850245.CEL	6	Low	40.78			0/0/7
RE13-000040-850236.CEL	4	Low	40.90	41.42	0.54	0/0/7
RE13-000041-850376.CEL	4	Low	41.38			0/0/7
RE13-000042-850395.CEL	4	Low	41.98			0/0/7
RE13-000043-850397.CEL	2	Low	41.23	41.17	0.70	0/1/7
RE13-000044-850358.CEL	2	Low	41.85			0/0/7
RE13-000045-850369.CEL	2	Low	40.45			0/0/7
			**Mean:**	**41.37**	**0.62**	
**Titration study experiment 2 Chip ID**	**μg aRNA hybridized**	**GEP70 risk group**	**GEP70 risk score**	**Mean GEP70**	**Standard deviation**	**Chip QC Metrics Fail/Warning/Pass**
RE13-000046-840059.CEL	10	Low	43.48	41.65	1.88	0/0/7
RE13-000047-840224.CEL	10	Low	41.76			0/0/7
RE13-000048-840239.CEL	10	Low	39.72			0/0/7
RE13-000049-840201.CEL	8	Low	43.95	43.46	0.43	0/0/7
RE13-000050-840219.CEL	8	Low	43.22			0/0/7
RE13-000051-840245.CEL	8	Low	43.20			0/0/7
RE13-000052-840243.CEL	6	Low	43.97	41.88	1.92	0/0/7
RE13-000053-840205.CEL	6	Low	40.19			0/0/7
RE13-000054-840242.CEL	6	Low	41.50			0/0/7
RE13-000055-840246.CEL	4	Low	42.71	43.20	0.96	0/0/7
RE13-000056-840051.CEL	4	Low	44.31			0/1/6
RE13-000057-840199.CEL	4	Low	42.59			0/0/7
RE13-000058-840213.CEL	2	Low	43.01	42.81	0.69	0/1/6
RE13-000059-840032.CEL	2	Low	42.04			0/1/6
RE13-000060-840236.CEL	2	Low	43.37			0/1/6
			**Mean:**	**42.60**	**1.18**	

These data also showed no significant changes in GEP data quality metrics across the range assessed. All hybridizations successfully passed the automated series quality control metrics developed by Signal Genetics, which are comprised of chip and data assessments (with associated pass/warning/fail thresholds) that have been established by analyzing large databases GeneChip quality data generated using aRNA concentrations at or above those recommended by the manufacturer [2].

Next we tested individual fresh or archival MM bone marrow aspirate samples submitted for MyPRS® analysis with RNA yields similar to those analyzed in the pooled-sample titration study. Twenty-two cases where the amount of either total RNA or aRNA obtained from the patients CD138+ place cells was insufficient for GeneChip hybridization were identified. These were hybridized using standard methods and the resulting GeneChip quality metrics were analyzed to refine the minimum RNA/aRNA thresholds necessary to generate a high quality GEP suitable for clinical use. As shown in Table 3, 12/13 specimens with > =3 ng/μL of total RNA and > =280 ng/μL aRNA resulted in successful hybridizations, defined as zero failed chip metrics and no more than three warning metrics). Below these thresholds a drop in hybridization quality was observed, indicating the GEP from such cases may be unreliable for clinical use.

**Table 3:** Low RNA-yield clinical specimens: Nanodrop RNA 260/280 ratio, aRNA concentration and GeneChip QC metrics

**Case number**	**Specimen type**	**Post-sort no. of CD138+ cells**	**RNA conc. (ng/μL)**	**aRNA conc. (ng/μL)**	**Chip QC Metrics (Fail, Warning, Pass)**	**Pass/Fail Chip QC**
RE13-000082	Archival samples with low yield	**36,000**	**11.0**	**1506.1**	**0,1,6**	**Pass**
RE13-000079	Archival samples with low yield	**32,000**	**5.3**	**708.6**	**0,1,6**	**Pass**
RE13-000080	Archival samples with low yield	**1,896,000**	**6.4**	**689.7**	**0,1,6**	**Pass**
RE13-000076	Archival samples with low yield	**40,000**	**3.1**	**545.1**	**0,1,6**	**Pass**
RE13-000092	Fresh sample with low yield	**22,000**	**10.4**	**533**	**0,0,7**	**Pass**
RE13-000074	Archival samples with low yield	**80,000**	**10.8**	**455.7**	**0,1,6**	**Pass**
RE13-000063	Archival samples with low yield	**76,000**	**8.1**	**426.4**	**0,0,7**	**Pass**
RE13-000077	Archival samples with low yield	**90,000**	**6.1**	**410.1**	**0,1,6**	**Pass**
RE13-000089	Fresh sample with low yield	**32,000**	**9.1**	**383.3**	**0,0,7**	**Pass**
RE13-000064	Archival samples with low yield	**180,000**	**5.5**	**339.9**	**0,1,6**	**Pass**
RE13-000073	Archival samples with low yield	**43,000**	**10.4**	**311.1**	**0,0,7**	**Pass**
RE13-000075	Archival samples with low yield	**80,000**	2.4	**304**	**0,1,6**	**Pass**
RE13-000066	Archival samples with low yield	18,000	**6.9**	**290.4**	**0,1,6**	**Pass**
RE13-000085	Fresh sample with low yield	**23,000**	**13.3**	**286**	**0,1,6**	**Pass**
RE13-000069	Archival samples with low yield	**24,000**	**8.3**	**281.1**	**0,1,6**	**Pass**
RE13-000088	Fresh sample with low yield	20,000	**9.6**	227.8	**0,1,6**	**Pass**
RE13-000081	Archival samples with low yield	**692,000**	**8.2**	**361.7**	3,2,2	Fail
RE13-000084	Fresh sample with low yield	**36,000**	**5.8**	**341.6**	1,0,6	Fail
RE13-000067	Archival samples with low yield	**188,000**	**6.2**	271.2	0,3,4	Fail
RE13-000068	Archival samples with low yield	**470,000**	**7.7**	263.3	2,1,4	Fail
RE13-000071	Archival samples with low yield	20,000	**7.7**	254.6	0,3,4	Fail
RE13-000061	Archival samples with low yield	**60,000**	**3.1**	220	0,3,4	Fail
RE13-000083	Archival samples with low yield	**192,000**	**5.4**	215.5	2,4,1	Fail
RE13-000078	Archival samples with low yield	17,000	**3.4**	214.6	1,2,4	Fail
RE13-000065	Archival samples with low yield	**36,000**	**5.5**	208.4	1,2,4	Fail
RE13-000090	Fresh sample with low yield	18,000	**4.8**	166.6	0,3,4	Fail
RE13-000091	Fresh sample with low yield	9,000	**6.2**	144.7	0,3,4	Fail
RE13-000062	Archival samples with low yield	10,000	2.4	122.8	2,3,2	Fail
RE13-000070	Archival samples with low yield	**30,000**	2.5	100.5	3,2,2	Fail
RE13-000086	Fresh sample with low yield	5,000	0.9	94.5	1,5,1	Fail

Values in bold type correspond to those passing the minimum acceptance threshold. Hybridization success is predicted using post-sort number of cells (>20,000) RNA concenctration (> = 3 ng/μL) and aRNA concentration (> = 280 ng/μL).

Consequently a threshold of > =3 ng/μL of total RNA and > =280 ng/μL aRNA was set for routine MyPRS testing. This amount of total RNA can be isolated from approximately 20,000 CD138+ plasma cells. After GeneChip hybridization, each profile must pass the ResultsPX GeneChip QC model before being used to calculate a patient’s GEP70 risk score, ensuring result integrity.

### Personalized gene expression heat-map assists in interpreting a patient’s GEP70 score in the context of patients with known outcomes

For each MyPRS® specimen analyzed, the 70 gene expression values used to compute the patients risk score are combined with a matrix of 70-gene data from 559 patients used to originally train and validate the prognostic algorithm [9] (data available at NCBI GEO ID: GSE2658). These gene expression profiles were generated from newly diagnosed patients who were enrolled in Total Therapy 2 (Thalidomide/Dexamethasone or Dex + high dose melphalan (Mel) supported autologous stem cell transplantation (ASCT)) or 3 (TT2; TT3; bortezomib-thalidomide-dexamethasone + Mel-ASCT) at UAMS prior to the commencement of their treatment.

In order to visualize the relationship between direction of differential gene expression and risk of relapse, hierarchical clustering is used to arrange the matrix rows (genes), while the columns (patients) are ordered by increasing GEP70 risk score. The published relapse-free-survival (RFS) times for patients in this trial are used to label each patient as <5 yrs RFS (blue) or >5 yrs RFS (red), allowing the physician to interpret the gene expression patterns in context with the end point of interest.

**Figure 5:**
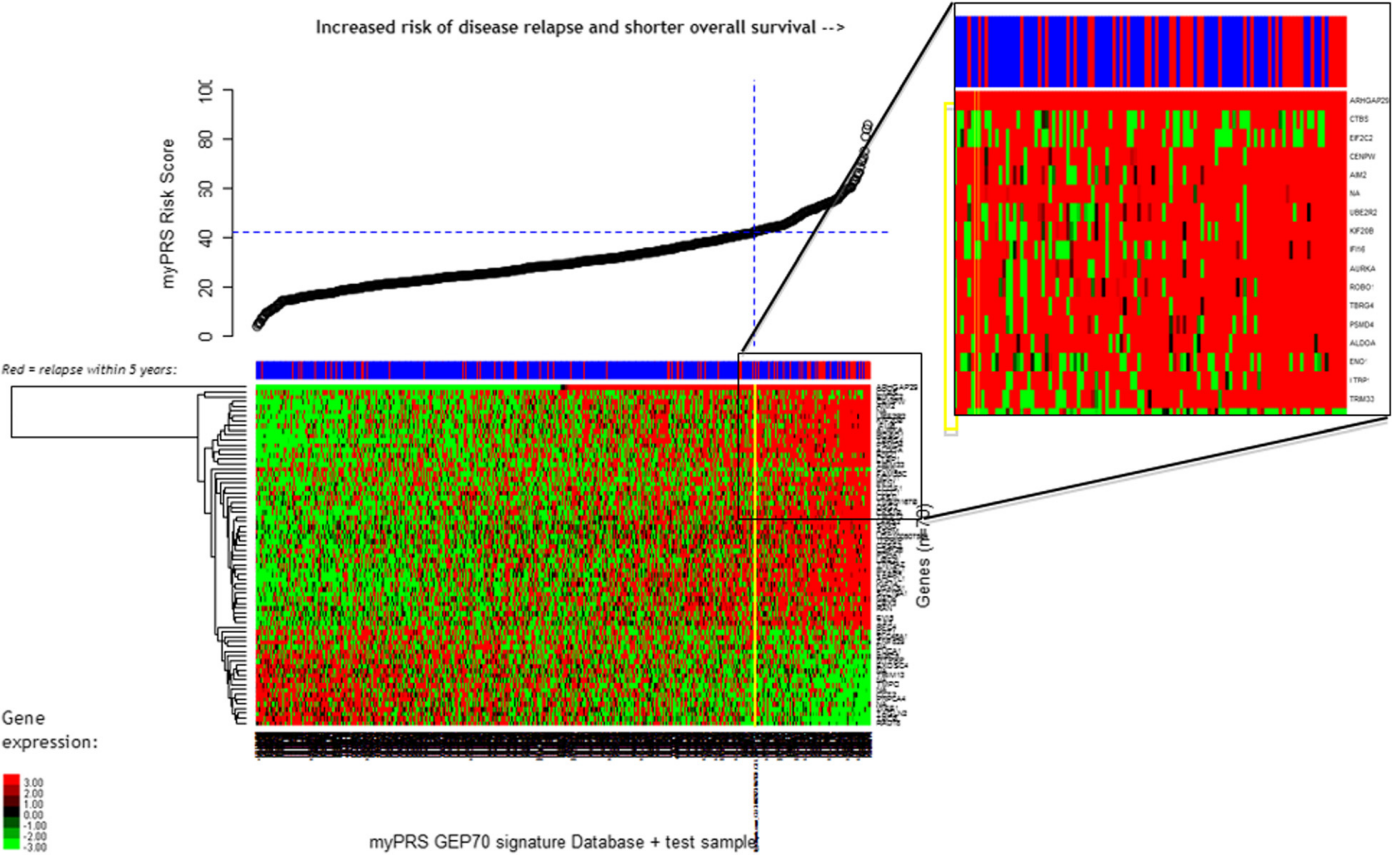
**Personalized MyPRS eene expression heatmaps; Generated for each MyPRS analysis performed to visualize the assocaition between the individual gene expression levels (green = low expression, red = high expression), GEP70 score and patient outcome.** Yellow line indicates the expression profile of the patient currently being analyzed, with the horizontal position determined by the individual GEP70 score. The red/blue panel at the top of the heatmap corresponds to 5 year relapse events, as observed in the algorithm training series.

As the example shown in Figure 5 illustrates, the GEP profile of the test patient is highlighted (yellow line), allowing the physician to visually compare the expression of the 70 prognostic genes in their patient compared to a large number of other multiple myeloma patients with known outcomes.

## Discussion

The UAMS 70-gene expression profile has been established as a powerful predictor of disease outcome in newly diagnosed and relapsed multiple myeloma patients. To enable use of this GEP algorithm in a high throughput clinical setting, a direct comparison of GEP70 scores generated in two laboratories was performed and minimum specimen requirements and quality control metrics were devised in order to ensure reliable, high quality prognostic results.

Outcome prediction was found to be highly similar for specimens analyzed in either the UAMS or Signal Genetics’ CLIA-certified laboratories. Importantly, a small number of cases in this study had discordant risk group predictions between the laboratories. These were cases where the risk score was very close to the classification threshold (45.2), indicating that care should be exercised when interpreting the risk score when it is close to this value. The number of cases at the threshold is exceedingly small as indicated by the bi-modal distribution of risk scores [6]. Further validation work was subsequently carried out to determine an appropriate confidence interval for the risk score, based on the observed technical noise present in the system.

The prognostic test was found to be highly stable over time as evidenced by the GEP70 scores from the MM cell line H929 control sample over a period of 12 months (Figure 2a). The 3% CV observed in these data over a twelve month period is similar to other microarray-based prognostic assays such as MammaPrint® (Agendia, CA.), a microarray based prognostic assay for breast cancer [27]. Analysis of more recent control sample data (late 2013- early 2014) shows further improvements to the assays consistency over time; CV 1.9% (Figure 2b). As a final and important observation from these control samples, no gradual shift in the risk score over time is detected, highlighting the long-term stability of the test.

Although the technical accuracy of MyPRS® is extremely high, samples close to the threshold have a higher chance of misclassification than samples further away from the threshold. However, the strong bi-modal distribution of scores proves that such cases are extremely rare. In principle, the chance of a patient with a poor clinical outcome incorrectly being assigned to a good prognosis profile should be minimized. Based on the known variation in the GEP70 risk profile, a small proportion of samples with indices close to the prediction threshold may be misclassified. For results that are close to the classification threshold of 45.2, it is recommended to evaluate the result in the context of the prognostic information present in the additional genomic signatures included in the MyPRS® assay; i.e. Molecular Subtype and Virtual Karyotype [9,28]. Additionally, a second sample from a separate anatomical site might be warranted. The implementation and validation of these additional signatures will be described in a separate publication.

By performing titration studies and analysis of low cellularity clinical specimens with RNA yields below manufacturer recommendations, we have determined that the Affymetrix GeneChip platform is able to generate high quality, reproducible gene expression profiles with RNA that can be isolated from as little as 20,000 CD138+ plasma cells in this context, even though the RNA yield is lower than the amount previously considered necessary. Multiple comparisons have shown the MyPRS® test is robust to the natural variation in clinical specimens submitted for analysis.

## Conclusion

GEP70 has been repeatedly demonstrated to be a statistically superior, standardized method of personalized multiple myeloma prognosis and molecular characterization, with less subjectivity than conventional methods such as FISH and cytogenetics. Even as the expression or mutational status of single genes are shown to influence response to specific myeloma treatments, e.g. expression levels of the glucocorticoid receptor gene NR3C1 and thalidomide [29], ‘treatment-independent’ risk-stratification assays such as GEP70 are likely to remain an important component in providing tailored treatment plans and ensuring optional outcomes.

The reproducibility of the MyPRS® test and the similarity of its results to those obtained from specimens analyzed in academic research laboratories demonstrate that it is an excellent tool to predict outcome of disease in MM patients. Standardization of specimen processing and the establishment of a comprehensive quality control program make the GEP70 assay highly suitable for the routine diagnostic clinical setting. Despite the wide variation in bone marrow aspirate specimen cellularity, we describe a series of novel quality control measurements that reliably produce high quality gene expression data, suitable for clinical use.

The MyPRS test is a stable, objective and standardized method for predicting prognosis in patients with multiple myeloma, supported by extensive clinical and technical validation data.

## Competing interests

RVL, RF, NB, JR, SR, and JDS are employees of Signal Genetics. BB and JDS hold patents on the use of gene expression profiling for multiple myeloma. RVL, SR and JDS own stock in Signal Genetics.

## Authors’ contributions

RF, NB, JR performed the specimen processing and gene expression profiling. RVL analyzed the quality control data and coordinated the drafting of the manuscript. SR, CH, BB and JDS were involved in drafting the manuscript, study design and coordination. All authors read and approved the manuscript.

## Pre-publication history

The pre-publication history for this paper can be accessed here:

http://www.biomedcentral.com/1755-8794/7/25/prepub
